# Generation of a Compendium of Transcription Factor Cascades and Identification of Potential Therapeutic Targets Using Graph Machine Learning

**DOI:** 10.3390/genes16121430

**Published:** 2025-11-30

**Authors:** Sonish Sivarajkumar, Romy Roy, Pratyush Tandale, Ankit Bhardwaj, Kipp W. Johnson, Anoop Titus, Benjamin S. Glicksberg, Shameer Khader, Kamlesh K. Yadav, Lakshminarayanan Subramanian

**Affiliations:** 1 Molecular Robotics, Kochi 682022, Kerala, India; sonish.sivarajkumar@gmail.com; 2 School of Computing and Information, University of Pittsburgh, Pennsylvania, PA 15213, USA; 3 Type 3 Civilization Technologies Pvt Ltd., Thiruvananthapuram 695013, Kerala, India; romyroy@t3ctec.com; 4 Health Informatics & Data Science, Georgetown University, Washington, DC 20007, USA; pratyushtandale@gmail.com; 5 Department of Computer Science, Courant Institute of Mathematical Sciences, New York University, New York, NY 10012, USA; bhardwaj.ankit@nyu.edu (A.B.); lakshmi@cs.nyu.edu (L.S.); 6 Institute for Next Generation Healthcare, Mount Sinai Health System, New York, NY 10065, USA; kipp.william.johnson@gmail.com; 7 Department of Medicine, Brown University, Providence, RI 02912, USA; anoop_titus@brown.edu; 8 Hasso Plattner Institute for Digital Health, Icahn School of Medicine at Mount Sinai, New York, NY 10029, USA; benjamin.glicksberg@mssm.edu; 9 Faculty of Medicine, Imperial College London, London SW7 2AZ, UK; 10 Department of Biomedical Sciences, College of Osteopathic Medicine, University of Northern Colorado, Greeley, CO 80639, USA

**Keywords:** transcription factors, TF cascades, graph machine learning, knowledge graph, therapeutic targets, pathway enrichment, cancer

## Abstract

Background: Transcription factors (TFs) are critical regulators of gene expression, and their dysregulation is implicated in diseases like cancer. This study aims to create a comprehensive resource of TF cascades to identify potential therapeutic targets. Methods: We extracted TF interactions from the STRING database, constructed a knowledge graph using graph machine learning, and performed pathway enrichment analysis with Enrichr. Network analysis and PageRank identified influential TFs. Results: We generated 81,488 unique TF cascades, with the longest containing 62 TFs. Key TFs (e.g., MYC, TP53, STAT3) were identified, and enriched pathways included cancer-related processes. A knowledge graph and dataset were made publicly available. Conclusions: This compendium of TF cascades provides a valuable resource for understanding TF interactions and identifying novel drug targets for precision therapeutics.

## 1. Introduction

Transcription factors (TF) are proteins that regulates the transcription of genetic information from DNA to RNA. Transcription factors bind to specific DNA sequences, called response elements, and help to control the expression of particular genes. They can either activate or inhibit the transcription of a gene, depending on the specific TF and the cell type in which it is functioning. TFs are important for the proper regulation of gene expression and are involved in many cellular processes, including development, cell growth and division, and response to environmental signals [1]. The key component of TFs is that they possess a DNA-binding domain; directly bind to DNA, which distinguishes them from other proteins, such as kinases, methylases, co-activators, histone deacetylases, histones acetyltransferases, and chromatin remodelers, that lack this domain; and act indirectly on gene expression. TFs are involved in regulating important pathways, such as immune responses [2] and cell type specification [3], and are used in laboratory experiments for cell differentiation [4], de-differentiation, and trans-differentiation [5].

**Figure 1:**
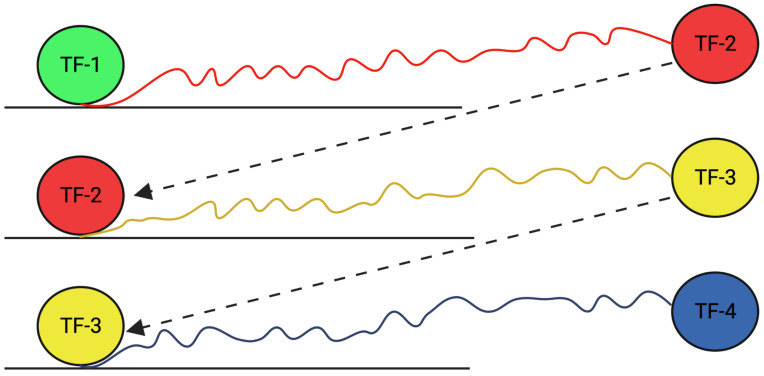
TF-1 binding to the DNA and activating TF-2, which induces the expression of TF-3 and in turn regulates TF-4, resulting in a cascade of TF production.

TFs can regulate gene expression by themselves or in cascades by activating the expression of a second TF, which then activates a third TF, resulting in the amplification of the original signal [6,7] (Figure 1). For this, the TF binds to specific DNA sequences and regulates another TF. This activated TF in turn goes on to regulate a third TF, creating cascades of gene expression. Thus, TF cascades refer to a series of TFs that work together to regulate gene expression. This cascade of events that leads to the regulation of a particular gene or set of genes results in the amplification of the initial signal and provides a regulatory relationship among TFs to maintain a high level of control over the expression of the target gene.

**Figure 2:**
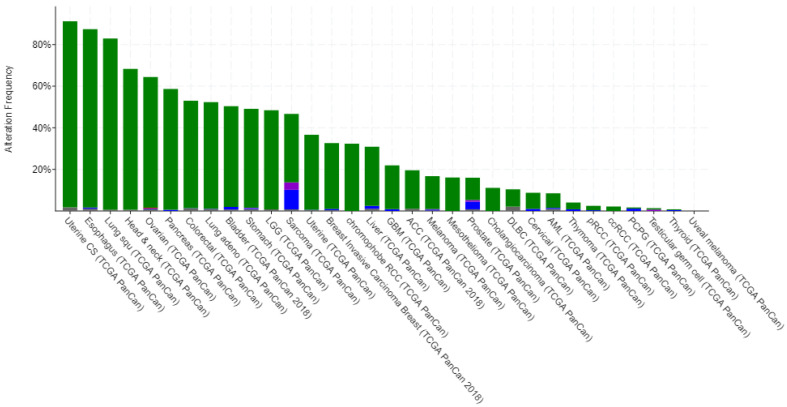
Alteration Frequency percentage vs. types of cancer bar graph plot for TP53 gene in human (developed using data from cBioPortal [10]).

TF cascades can be disrupted by mutations in the genes that encode the TFs or by changes in the regulatory elements that control their expression. Dysregulation of TF cascades can lead to abnormal gene expression and can contribute to the development of diseases such as cancer. For example, one of the most commonly known and most frequently mutated TFs in cancer is TP53 [8]. There have been numerous clinical data records showing that TP53 is found to be mutated in the majority of types of cancer as shown in (Figure 2).

Thus, TF cascades are important for the proper regulation of gene expression and are involved in many cellular processes, including development, cell growth and division, and response to environmental signals. They allow cells to respond to specific signals in a highly coordinated and controlled manner. For example, when a cell receives a signal from its environment, a TF cascade may be activated, leading to the expression of specific genes that allow the cell to respond to the signal.

TF cascades can be disrupted by mutations in the genes that encode the TFs or by changes in the regulatory elements that control their expression. Dysregulation of TF cascades can lead to abnormal gene expression and can contribute to the development of diseases such as cancer. Mutations in TFs can cause cancer, as in the case of one of the most commonly known and most frequent mutations of a TF, TP53 [9]. There have been numerous clinical data records showing that TP53 is mutated in the majority of types of cancer, as shown in Supplementary Figure S1.

Thus, despite their potential role in cancer therapy, genome-scale TF cascades have remained largely unexplored and underutilized. In fact, due to the lack of information and appreciation for their role in cancer treatment to date, not many studies have identified TF cascades as therapeutic targets. This has created a significant knowledge gap and a missed therapeutic opportunity. Therefore, there is an urgent need to compile a comprehensive and high-quality resource on genome-scale TF cascades and their associated pathways, which can facilitate the discovery of novel drug targets and biomarkers. Moreover, such a resource can enable the application of advanced analytics and AI approaches, such as network analysis, knowledge graphs, and graph machine learning, which can capture the complex and dynamic interactions among TFs and genes, and provide more accurate and robust predictions than conventional methods [11]. A resource on genome-scale TF cascades can aid research in cancer genomics and therapeutics.

Our study leveraged multiple techniques, including network analysis, concepts from knowledge graphs, and graph machine learning, to analyze TF cascades and their role in various diseases. Specifically, we began with TF-pathway enrichment analysis to identify relevant pathways and generate a list of candidate genes. Next, we constructed a comprehensive representation of the interactions between TFs and human biological pathways. To achieve this goal, we incorporated data from multiple sources and constructed a TF cascade graph to capture the interactions within TFs and between TFs and biological pathways. Through the application of network analysis and graph machine learning techniques, we were able to identify key regulatory relationships and predict novel interactions, enabling us to uncover potential therapeutic targets and biomarkers for further exploration. Overall, our study highlights the power of integrating multiple approaches to tackle complex biological problems and provides a valuable resource for researchers working in the field of drug discovery and precision therapeutics.

The use of graph-based methods has emerged as a promising approach for predicting drug target interactions and therapeutic interactions with biomarkers [12,13]. Recent studies have demonstrated that graph database methods using knowledge graphs and graph machine learning can be used to represent biological entities and relationships, and enable entity target interaction studies [14,15]. Our proposed TF cascade graph and dataset provide a comprehensive and high-quality resource for researchers to explore the intricate interactions between TFs and genes, leading to the identification of new therapeutic targets and biomarkers. By integrating deep learning and graph-based approaches, we can overcome the limitations of previous methods and gain more profound insights into the underlying biology of diseases. Consequently, the impact of our TF cascade graph and dataset will be far-reaching, facilitating drug discovery and development, and ultimately improving patient outcomes.

## 2. Data and Methods

### 2.1. Data

#### 2.1.1. STRING Database

STRING (Search Tool for the Retrieval of Interacting Genes/Proteins) is a database of protein protein interactions that includes both physical interactions (such as those mediated by direct physical contact between proteins) and functional associations (such as those mediated by shared protein function or shared regulation of gene expression) [16]. The database includes interactions from a wide variety of sources, including high-throughput experiments, computational predictions, and manual curation from the scientific literature.

STRING is a useful resource for researchers who are interested in understanding the relationships between proteins and how they function in cells. It can be used to identify potential protein-protein interactions, predict the functions of proteins, and understand how proteins function in the context of larger networks. The database is also useful for studying the relationships between proteins and diseases, as disruptions in protein-protein interactions can contribute to the development of diseases such as cancer.

In addition to protein-protein interactions, STRING also includes information on small molecules and their interactions with proteins. It also includes functional annotation for proteins, including information on their pathways, functions, and structural features. The database is constantly being updated with new information, and it is widely used by researchers in the field of biology and biomedicine.

#### 2.1.2. TISSUES Database

TISSUES is a web resource updated weekly that integrates evidence on tissue expression from the manually curated literature, proteomics and transcriptomics screens, and automatic text mining [17]. They map all evidence to common protein identifiers and Brenda Tissue Ontology terms, and further unify it by assigning confidence scores that facilitate comparison of the different types and sources of evidence.

#### 2.1.3. cBioPortal

cBioPortal is a database which contains patient cancer data, including clinical and genomic data [10]. It has complete details about patients in a complete and modular fashion, where you can choose to download specific parts of the patent data, i.e., genes with mutated data, patient race, patient age, etc.

### 2.2. Methods

#### 2.2.1. TF Interaction Extraction

Directionality and cascade assembly. We used the STRING v11 actions human dataset to derive directed TF→TF edges with the mode of regulation (activation/inhibition).

Only TF pairs where both proteins were in our curated TF list were retained and edges were oriented from regulator to target. We required a combined score ≥700 (high confidence); interactions present only in undirected PPI layers or lacking curated TF→TF regulation (e.g., in TRED/TRANSFAC) were excluded from cascade construction. We constructed a directed graph G=(V,E) over TFs and enumerated simple directed paths using depth-first search from source nodes (in-degree =0), pruning cycles and deduplicating node sequences. Chain length is the number of TFs in a path and cascade level *L* is chain length−1. We have released the retained TF→TF edges with scores and the path-enumeration parameters for full reproducibility.

The STRING database was used to obtain information on all protein interactions. This database encompasses all known interactions between approximately 5090 species. For the analysis, only the human action data from version 11 of the database was extracted. To focus solely on transcription factors (TFs) among all the proteins, a list of TFs was required. This list, which was published earlier [1], is currently hosted on the University of Toronto’s server. Subsequently, the interaction data of TFs were gathered by selecting known TFs from the human protein dataset.

We mapped the TF list from Ensembl gene identifiers to UniProt protein identifiers [18] using BioMart (Human dataset), enabling consistent integration with STRING protein interactions.

Tissue data was extracted to determine the location of each TF. The data was acquired from the TISSUES database. We have limited our study to cancer studies from cBioPortal. The data was fetched using a script from the *cgdsr* R package v1.3.o [19]. This package has functions, which are used for querying the Cancer Genomics Data Server (CGDS, http://www.cbioportal.org/datasets, accessed on 1 January 2020), which is hosted by the Computational Biology Center at MSKCC.

#### 2.2.2. Building the TF Cascade Dataset

**Figure 3:**
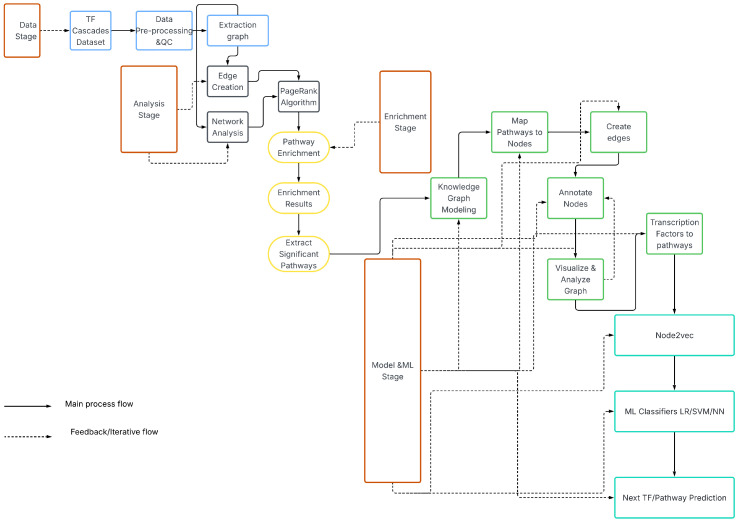
Overall analytics and ML workflow.

The process of preprocessing the TF interaction data was crucial to ensure that the data is accurate and reliable when building TF cascades. We removed duplicate TF interactions to eliminate any redundant information that existed in the dataset. Additionally, we formatted the data in a way that could be easily used for building the TF cascades; i.e., we organized the data so that it could be easily read and analyzed by the algorithms that were used to build the cascades.The Overall workflow is depicted on (Figure 3).

The obtained cascades can be filtered out based on the size or specific requirements to obtain a more specific and informative result. We also reindexed the dataset by adding the cascade level to each of the cascades. For instance, TF 1 -/- > TF 2 -/- > TF 3 were assigned a length of 3, indicating three TFs are present in the chain, but with L2 as the cascade level as they have two levels of cascades.

#### 2.2.3. Analytics

##### Cascade Summary

Note on sparsity/missingness: Cells for deeper TF positions are structurally missing when cascades are shorter; centrality is computed on the extracted directed TF graph and is unaffected by such NA placeholders.

TF cascades refer to linear or branching pathways of gene regulation, where the expression of one TF can influence the expression of downstream TFs. We performed Exploratory Data Analysis (EDA) to assess and summarize the database’s primary characteristics, as it is difficult to examine each element of a dataset and select the most relevant ones. Our analysis was performed using the python library *pandas-profiling* and employed different visualization methods to discover the underlying patterns in the data, enabling us to comprehend what the data is communicating. Several graphical and non-graphical analysis techniques were employed to analyze the TF cascade data. We restricted our investigation to univariate analysis because we are primarily interested in categorical variables TFs.

##### Page Rank

*PageRank* is an algorithm developed by Google to rank web pages in their search engine results based on the number and quality of links pointing to them [20]. The algorithm is based on the idea that a web page is more important if it is linked to by other important pages, and less important if it is linked to by less important pages. PageRank has been widely used and studied in the field of information retrieval and has inspired the development of similar algorithms in other fields.

We applied the PageRank algorithm in the TF cascades dataset to rank the TFs based on their connectivity and centrality within the cascade. TF cascades can be represented as directed graphs, with TFs represented as nodes and regulatory interactions represented as edges.

We computed PageRank on the directed TF network (damping = 0.85) and used the scores to prioritize convergence nodes across cascades. The PageRank algorithm provided valuable insights into the functional roles and relationships of the TFs in a TF cascade.

##### Extraction Graph

TF cascades can be represented as directed graphs, where the TFs are represented as nodes and the regulatory interactions between them are represented as edges. The direction of the edges reflects the direction of the regulatory influence, with the upstream TFs regulating the downstream TFs. This will facilitate a better understanding of the TFs with the highest scores (represented by the graph’s nodes). This will allow us to focus on the most important TFs in terms of medication development and personalized medicine.

For example, consider a simple TF cascade with three TFs, A, B, and C, where A regulates B, and B regulates C. This cascade can be represented as a directed graph with three nodes (A, B, C) and two edges (A > B, B > C). The direction of the edges reflects the regulatory relationships between the TFs, with A regulating B and B regulating C. The graph representation of a TF cascade captures the hierarchical structure and relationships between the TFs and can be used to analyze and understand the regulatory mechanisms of the cascade.

The graph representation of a TF cascade can be constructed from experimental or computational data on the regulatory interactions between the TFs. There are various databases and resources available that provide information on transcriptional regulatory interactions, such as TRED and Transfac. In addition to the regulatory TF interactions, other information, such as the associated biological pathways and timing of the regulatory pathways, can be incorporated into the graph representation to provide a more comprehensive view of the TF cascade, which is conducted in the later part of the study. The graph representation of a TF cascade was analyzed using various network analysis algorithms and tools to identify patterns and trends in the regulatory network and to gain insights into the underlying regulatory mechanisms.

##### Network Analysis on the Extraction Graph

Network analysis is a powerful approach for exploring and understanding the relationships between genes and pathways in the context of complex biological systems [12,21,22]. In the context of TF cascades, network analysis can be performed on the directed graph representation of the TF cascade to gain insights into the functional connections and interdependencies between the TFs and the pathways of transcriptional regulation.

Centrality measures are a class of network analysis metrics that are used to identify the most central or influential nodes in a network. There are several types of centrality measures, including degree centrality, betweenness centrality, and eigenvector centrality.

Degree centrality measures the number of connections a node has in the network. In the context of a TF cascade, degree centrality can be used to identify the TFs that have the most regulatory interactions with other TFs. These TFs may be central to the functioning of the cascade and may be potential therapeutic targets.

Betweenness centrality measures the number of shortest paths between pairs of nodes that pass through a given node. In the context of a TF cascade, betweenness centrality can be used to identify the TFs that are most central or influential in the cascade, as they are likely to be involved in multiple regulatory pathways and may have a broad impact on the cascade.

Eigenvector centrality measures the influence of a node based on the influence of its neighbors. In the context of a TF cascade, eigenvector centrality can be used to identify the TFs that are most central or influential in the cascade, based on the collective influence of their regulatory targets. TFs with high eigenvector centrality are likely to have a broad impact on the cascade and may be potential therapeutic targets.

##### Pathway Enrichment

We conducted gene set enrichment analysis for the purpose of functional investigation of TF cascades and identification of disease phenotypes. Pathway enrichment analysis is a common approach used in bioinformatics to identify pathways or biological processes that are significantly enriched in a given gene set. This is often done to understand the underlying biological mechanisms or functions associated with a particular set of genes, such as those differentially expressed in a disease state or in response to a particular treatment.

We used the Enrichr tool [23] to perform pathway enrichment analysis on a set of 500 human TFs. Enrichr is a widely used online tool which allows users to input a list of genes and returns a list of significantly enriched pathways and functional categories based on the gene set. Enrichr uses a large number of databases and resources, including KEGG, Reactome, Gene Ontology, and others, to provide a comprehensive coverage of pathways and functional annotations.

We performed enrichment with Enrichr on TF sets and report Benjamini Hochberg [24] FDR-adjusted *p*-values (α=0.05).

The results of pathway enrichment analysis could provide valuable insights into the biological functions and mechanisms associated with the extracted TF set [25].

##### Knowledge Graph Modeling

A knowledge graph is a structured representation of knowledge that captures the relationships between entities and their attributes. We constructed a knowledge graph on the results of pathway enrichment analysis to capture the relationships between the enriched pathways and the TFs in the cascade.

From the pathway-enriched TF cascade dataset, we identified the significantly enriched pathways in the TF cascade, based on the adjusted *p*-value threshold of 0.05. Next, we filtered the genes in the enriched pathways and mapped them to the corresponding nodes in the directed graph representation of the TF cascades. We modeled the edges between the enriched pathways and the corresponding nodes in the directed graph to capture the relationships between the pathways and the TFs. The edges were created by linking the enriched pathways, represented as nodes, to the corresponding TFs, represented as nodes in the directed graph (extraction graph). The nodes and edges were then annotated with additional attributes, such as gene names, pathway names, and regulatory relationships [26]. The nodes were annotated with the gene names and the pathways were annotated with their names and descriptions. We also embedded the cascade levels in each cascade as the attribute of the TF nodes. Finally, we created a NetworkX visualization [27] of the resulting knowledge graph to gain insights into the relationships between the enriched pathways and the TFs in the cascade.

The knowledge graph on the TF cascades provided a structured and interactive representation of the relationships between the enriched pathways and the TFs in the cascade. It was then used to explore and analyze the relationships between the pathways and the TFs and to understand the functional roles and relationships of the TFs within the cascade. The knowledge graph can also be used to identify potential therapeutic targets and mechanisms of action and to inform further experimental studies.

The purpose of creating a graph from the newly constructed TF cascades and enriched pathway dataset is to establish a more comprehensive and generalizable representation of TF cascades. This approach enables us to construct a foundational model that can be readily applied to various downstream tasks, such as Next TF prediction, TF classification, and Pathway prediction, among others. By doing so, we ensure that researchers in this field have access to a complete resource that includes the dataset as well as the foundational model. Consequently, this approach can serve as a valuable tool to promote and advance further research in this area. Moreover, graph approaches are preferred over Recurrent Neural Network (RNN) and Hidden Markov Model (HMM) approaches when we are interested in the relationships between entities rather than sequences of states of a single entity. The TF cascades problem fits the graph paradigm better because different TFs are different entities that have regulatory relationships between them, and not a single entity showing different sequences of states. Therefore, our graph-based model can capture the complex and dynamic interactions among TFs more effectively than other methods.

##### Graph Machine Learning (Graph ML) Tasks That Enable Biological Discovery

Graph machine learning has emerged as a promising approach for analyzing heterogeneous biological data by capturing complex relationships between entities [28]. Common graph machine learning tasks include link prediction, which can be used to predict protein protein interactions or drug target interactions, and node classification, which can predict the function of a protein or the type of a cell. Graph embeddings can also be learned to provide low-dimensional vector representations for each node in the graph, which can be used for downstream tasks [28].

TF prediction Link prediction is a technique that can be applied to predict missing or future links in a network, such as relationships between genes or pathways in a biological network. We employed link prediction methods to predict the next TF in a given cascade, based on the relationships between the existing TFs in the cascade. This approach is based on the idea that nodes with similar attributes are more likely to be linked in the future. The cascade levels of TFs in the cascade, which were used as attributes for the nodes, were considered as the expression level of a TF and a similarity measure (Cosine similarity) was applied.

**Figure 4:**
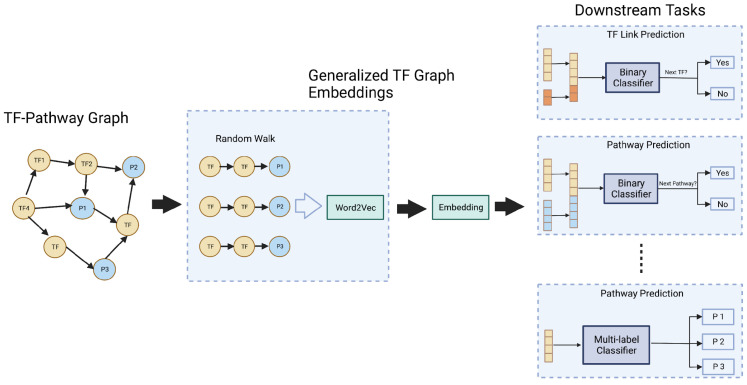
Node2vec based graph embedding workflow diagram.

For performing this similarity-based TF link prediction in a given cascade, we employed a network embedding technique called Node2vec [29]. The Node2vec algorithm was used to learn low-dimensional representations of the nodes in the enriched TF cascade KG, which could capture the network’s topology and could preserve the structural relationships between nodes. The cosine similarity measure was then applied to the learned TF cascade pathway embeddings to predict the next TF in a given cascade (Figure 4). It is important to note that Node2vec can handle large and sparse networks and can also capture higher-order relationships, which makes it suitable to be used as a TF link prediction method.

To obtain a link embedding from the node embeddings, we applied different operators such as Hadamard, L1, L2, and average to combine them. These operators are applied to combine the embeddings of the two nodes to form the resulting link embedding. The Hadamard operator, also known as the element-wise multiplication operator, takes the element-wise product of the embeddings of two nodes, resulting in a link embedding with the same dimensionality as the input node embeddings. Mathematically, for two embeddings u and v, the Hadamard operator is defined as u⊙v. The L1 operator, also known as the Manhattan distance operator, takes the absolute difference between the embeddings of two nodes, resulting in a link embedding with the same dimensionality as the input node embeddings. Mathematically, for two embeddings u and v, the L1 operator is defined as |u−v|. The L2 operator, also known as the Euclidean distance operator, takes the square root of the sum of the squared differences between the embeddings of two nodes, resulting in a link embedding with the same dimensionality as the input node embeddings. Mathematically, for two embeddings u and v, the L2 operator is defined as $\sqrt{{\left(u-v\right)}^{2}}$.

The average operator takes the average of the embeddings of two nodes, resulting in a link embedding with the same dimensionality as the input node embeddings. Mathematically, for two embeddings u and v, the average operator is defined as (u+v)/2. These operators have different implications for link prediction performance, depending on the characteristics of the graph and the specific task at hand. The next step was to evaluate and rank the predicted links based on the scores generated by the link prediction algorithm.

Pathway prediction is another experiment which we performed using the pathway-enriched TF cascade knowledge graph. The process can be similar to the steps used for predicting the next TF in a cascade, but instead of predicting links between TFs, the focus would be on predicting links between pathways and TFs.

TF cascade link prediction is a powerful approach but it cannot replace experimental validation and functional assays, as it would be crucial to validate TF predictions experimentally to ensure their accuracy, especially for therapeutic applications. It is important to mention that link prediction is a challenging task, and the performance of link prediction methods can be affected by the sparsity and the size of the network. It is also crucial to validate the predictions experimentally. The TF or pathway predictions can be tested by perturbing the genes in the network using gene editing techniques or other methods and observing the changes in the network’s topology.

In addition to next-TF or pathway prediction, the following approaches can also be used to identify potential therapeutic targets using the TF cascades dataset and knowledge graph:

- Identifying Key TFs: The knowledge graph can be analyzed to identify the TFs that are central or influential in the cascade, based on their connectivity and centrality within the network. These TFs may be central to the functioning of the cascade and may be potential therapeutic targets. For example, by using centrality measures like degree centrality, betweenness centrality and eigenvector centrality on the knowledge graph, one can identify the TFs that have the most regulatory interactions with other TFs, or the TFs that are most central or influential in the cascade, as they are likely to be involved in multiple regulatory pathways and may have a broad impact on the cascade.
- Identifying Enriched Pathways: The knowledge graph can be analyzed to identify the enriched pathways that are most significant or relevant to the cascade, based on their *p*-value or other statistical measures. These pathways may be involved in the cascade and may be potential therapeutic targets.
- Network Analysis: One can use various network analysis techniques like clustering, modularity, centrality, etc., to identify the modules and subnetworks of the knowledge graph, which may represent the functional pathways and mechanisms of action within the cascade.
- Identifying Regulatory Relationships: The knowledge graph can be analyzed to identify the regulatory relationships between the TFs and the pathways, and the mechanisms of these relationships. For example, by looking at the edges and annotation on the edges in the knowledge graph, one can identify the type of regulatory relationship (e.g., activation, repression) and any additional information about the regulatory mechanisms (e.g., binding sites, motifs).
- Validation: The potential targets and mechanisms identified using the knowledge graph can be validated using experimental data. This can be achieved by performing experiments such as gene knockdown or overexpression, or by analyzing gene expression data from relevant biological contexts. Validation can provide valuable insights into the biological functions and mechanisms of action of the potential targets.

## 3. Results

### 3.1. TF Cascades Dataset

In this study, we aimed to create a compendium of TF cascades from the dataset of TF interactions extracted from the STRING database. The raw human protein interaction data obtained from the STRING database contained a total of 11 million interactions. To focus specifically on transcription factors, the dataset was pruned by removing all non-TF proteins, resulting in a reduced dataset of 300,000 interactions.

The whole protein ID TF cascade was converted to gene ID TF cascades by creating a dictionary using BioMart Ensembl release v.115, where one column contained all the Protein IDs and the second column contained their respective gene names, as shown in Table 1. The TF cascades were converted to gene cascades using a Python script.

**Figure 5:**
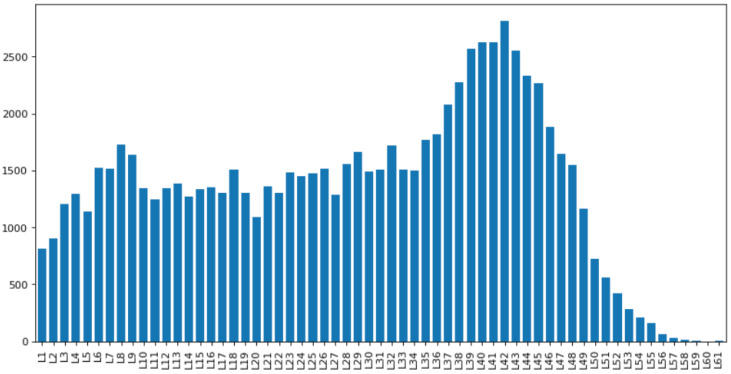
Frequency distribution of TF cascades in each cascade level.

Tissue data was then taken and filtered to keep only proteins of interest. Cancer patient data was taken from cBioPortal, and genes which showed mutation(s) were cross-checked with the genes which were present in our cascades. From the TF cascades built, we identified 81,488 unique TF cascade chains, where the longest chain had a length of 62 TFs (cascade level L61, (Figure 5). having only 426 unique genes out of 1636 total present in humans. The cascades were plotted according to different lengths (Figure 5), where cascades with length 43 had the highest frequency of 2815. We also found more than 2500 occurrences for TF cascade levels from L39 to L43.

**Figure 6:**
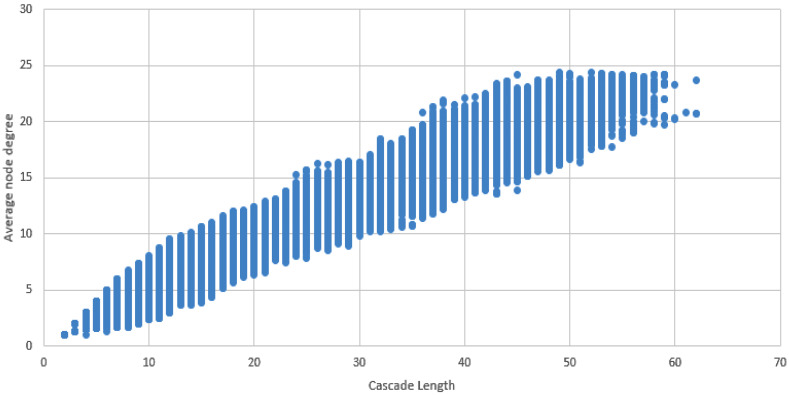
Dot plot displaying average node degree vs. length of the cascade.

These results indicate a complex network of interactions between TFs, and suggest that multiple TFs work together to regulate gene expression. The high number of unique TF cascades (81,488) highlights the intricate nature of these interactions. Furthermore, the length of the longest cascade (L61) indicates the scale of these regulatory networks(Figure 6).

### 3.2. Summary of TF Cascades Dataset

We created a website to show the EDA results to researchers interested in using the TF cascades dataset (https://sonishsivarajkumar.github.io/TFCascades/ accessed on 28 August 2025).

The Exploratory Data Analysis (EDA) of the TF cascades dataset provided valuable insights into its primary characteristics. The dataset consists of 64 variables (cascade id, cascade level and 62 TFs) and 81,488 observations.

The index variable is a real number that assigns a cascade ID, ranging from 1 to 81,488. It is essential for indexing the cascades and assigning cascade levels. The “Level” variable, which represents the cascade levels, is a categorical variable with 61 distinct values, indicating that the cascades have different levels of complexity. The most frequent cascade levels are Level 42, Level 41, Level 40, Level 39, and Level 43. These levels are present in 2815, 2628, 2625, 2570, and 2548 cascades, respectively. The remaining cascade levels have a total count of 68,302 cascades.

The “TF 1” variable represents the first TF in each cascade and also exhibits high cardinality. It has 311 distinct values, with STAT3 being the most frequent TF present in 3419 cascades. Other frequently occurring TFs include POU5F1 (1546 cascades), TP53 (1371 cascades), SOX2 (1166 cascades), and FOXO3 (1157 cascades). The remaining TFs have a total count of 72,829 cascades. Similarly, the “TF 2” variable represents the second TF in each cascade and has 291 distinct values. The most frequent TF in this variable is MYC, present in 4231 cascades. TP53 (2821 cascades), NANOG (2687 cascades), AR (2091 cascades), and FOXP3 (2,050 cascades) are also high-occurring TFs. The remaining TFs have a total count of 67,608 cascades.

The ’TF 3’ variable, representing the third TF in each cascade, has 233 distinct values. The most frequent TF in this variable is NANOG, present in 12,659 cascades. SNAI1 (8178 cascades), TP53 (4448 cascades), POU5F1 (4385 cascades), and PPARG (2670 cascades) are also prominent TFs. There are 813 missing values in this variable. The remaining TF variables (TF 4 to TF 62) exhibit a similar pattern, with varying numbers of distinct TFs and missing values. These variables have high cardinality and missing values ranging from 2.1% to 39.1%.

The EDA provided a comprehensive summary of the dataset, highlighting the characteristics of the TF cascades. The dataset contains a diverse set of TFs, each involved in various cascade levels. However, missing values and high cardinality pose challenges in the analysis and interpretation of the data. Further investigations and statistical techniques were applied to uncover additional insights from this TF cascades dataset.

### 3.3. PageRank Results

After running 10 iterations of PageRank with cascade connection serving as the initial weights of all nodes set to 0.25, the analysis revealed that the *NANOG* gene obtained the highest ranking with a score of 0.016963, while the *TP53* gene was ranked second with a score of 0.011583 Table 2.

### 3.4. Network Analysis of the Extraction Graph

The TF cascades extraction graph was built by combining all the cascades into a single graph. The network comprised 426 nodes representing 426 unique TFs, and 866 edges which correspond to 866 interactions between all the TFs. From the detailed analysis of the network, we identified 10 TFs which had the highest regulatory influence through three centrality measurements betweenness centrality, closeness centrality, and eigenvector centrality as shown in Table 3, Table 4 and Table 5.

MYC, TP53, and STAT3 were the top three TFs based on all three centrality measurements. In terms of betweenness centrality, MYC had the highest score of 0.1694, followed by TP53 with a score of 0.1464 and STAT3 with a score of 0.1378. Based on closeness centrality, MYC had the highest score of 0.3538, followed by STAT3 with a score of 0.3534 and TP53 with a score of 0.3481. Based on eigenvector centrality, STAT3 had the highest score of 0.3665, followed by MYC with a score of 0.3037 and TP53 with a score of 0.2606.

The top three TFs identified in this study, MYC, TP53, and STAT3, are well-known regulators of various cellular processes, including cell growth, proliferation, and apoptosis. MYC is a proto-oncogene that plays a critical role in regulating cell growth and proliferation. TP53 is a tumor suppressor gene that is involved in regulating apoptosis and DNA repair. STAT3 is a TF that plays a crucial role in regulating immune responses and cell proliferation. The identification of these TFs as the top regulators in the network supports their importance in regulating cellular processes.

#### Analyzing the Prognostic Relevance of POU5F1

We prioritized TFs for clinical correlation if they satisfied the following: (i) top-decile centrality across at least two measures (PageRank, eigenvector, closeness or betweenness); (ii) presence in ≥1000 cascades; and (iii) non-zero alteration frequency across multiple pan-cancer cohorts in cBioPortal. POU5F1 met all three criteria and was selected as a representative case.

The network analysis of the TF cascades dataset identified POU5F1 as a key node. The TF POU5F1, known as OCT4, plays a crucial role in maintaining the essential characteristics of Embryonic Stem Cells (ESCs), such as their ability to renew themselves and their pluripotency, which is the capacity to develop into different cell types [30,31]. Together with other key transcription factors, SOX2 and NANOG, POU5F1 activates specific genes that are vital for maintaining ESCs’ unique properties [30]. This group of factors works by binding to specific DNA regions that control gene activity, thus maintaining the cells’ identity and potential for differentiation. Alterations in the function or levels of POU5F1 can disrupt these processes and may contribute to the development of cancer, as this gene can act as an oncogene when improperly regulated. Given its central role in cell identity and proliferation, POU5F1 is a subject of intense study, not only for its fundamental role in cell biology but also for its potential as a target in cancer treatment strategies.

**Figure 7:**
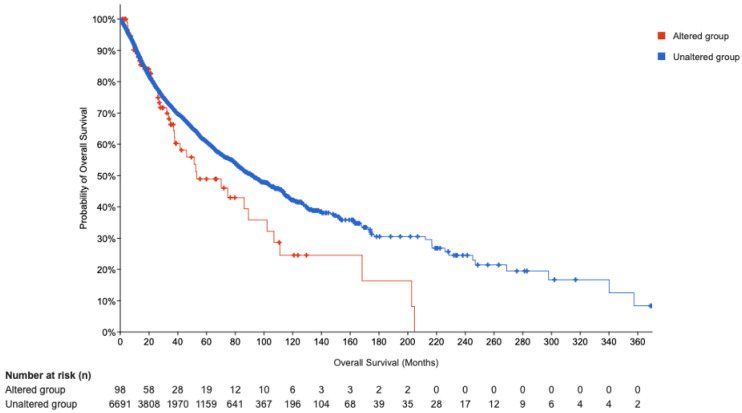
KM curve.

Subsequently, we performed Kaplan Meier (KM) survival analyses (Figure 7). which have provided valuable insights into the clinical significance of POU5F1 alterations in patient outcomes. The disease-specific survival analysis encompassed 6553 patients, revealing a statistically significant disparity in survival outcomes based on POU5F1 status, with a *p*-value of 0.0041 and a q-value of 0.0168 (Table 6). This significance persisted even after adjusting for multiple survival comparisons, strengthening the evidence that POU5F1 alterations are indeed important. Furthermore, the overall survival analysis, including 6789 patients, corroborated these findings with a *p*-value of 0.0318 and a q-value of 0.0437. Progression-free survival presented a similar pattern, suggesting a robust association between POU5F1 alterations and decreased survival metrics. In contrast, disease-free survival analysis of 3,736 patients did not exhibit a significant difference between altered and unaltered POU5F1 groups, indicating the specificity of POU5F1’s impact on survival post-treatment.

The survival plot for the altered group, with 98 cases and 43 events, delineated a median overall survival of 53.19 months, substantively lower than the 91.99 months observed in the unaltered group of 6691 patients (Table 7). This substantial reduction in median survival time for patients with altered POU5F1 expression highlights the transcription factor’s potential role as a prognostic marker.

The findings delineated above validate our initial hypothesis that POU5F1 alterations could portend unfavorable clinical outcomes. Given the pivotal role of POU5F1 in the regulatory networks of gene expression, its dysregulation may serve as a critical indicator of disease progression and patient prognosis. The statistical rigor of the KM survival analysis lends weight to the proposition that POU5F1 could be a viable target for therapeutic intervention, particularly in conditions where its expression is aberrant.

The confluence of network analysis and KM survival analysis in this study provides a compelling narrative about the role of POU5F1 in disease etiology and progression. It underscores the utility of this new dataset in unearthing potential therapeutic targets, with POU5F1 emerging as a TF of considerable interest for future research and clinical exploration.

### 3.5. Pathway Enrichment

Our pathway enrichment analysis provided valuable insights into the biological functions and mechanisms of action of TFs. The enrichment analysis resulted in functional categories that are significantly enriched in the input gene set, which were ranked by the adjusted *p*-value. The adjusted *p*-value is calculated using the Benjamini Hochberg method to control for the false discovery rate. In addition to the list of enriched pathways, the enrichment analysis provided a variety of other information and resources, such as gene gene interactions and odds ratios.

The results of pathway enrichment analysis using Enrichr revealed significant enrichment of various pathways and functional categories among the TF gene set. For the 81,488 cascades, we obtained 2 million pathways with an average of 25 pathways for each cascade (Table 8). The top enriched pathways included “Transcriptional misregulation in cancer,” “gnrh signaling pathway,” and “Th17 cell differentiation,” which are known to be involved in cancer and various other diseases. Other enriched pathways included “neuroactive ligand receptor interaction,” “adipocytokine signaling pathway,” and “hedgehog signaling pathway,” which are involved in development, differentiation, and cell signaling.

### 3.6. Knowledge Graph

The knowledge graph was constructed as a directed multigraph with a total of 9784 nodes and 38,627 edges (Table 9). The node types present in the graph include only a single type, i.e., source, which represents the TFs in the TF cascade. Each TF node points towards the next TF node in the cascade and also towards the pathways, which are the targets in this graph. The graph is node-labeled and attributed, where each node contains information about the *p*-value, Z-score, and combined score for all the available pathways.

Furthermore, the edges in the graph are unlabelled but attributed, and each edge is directed from the source node to the target node (source → target). The weights of the edges range from 1.13 × 10^−19^ to 0.437467, with a mean of 0.154782 and standard deviation of 0.114678 (Table 10). The graph can be used to represent the relationships between the TFs and pathways in the cascade and can be leveraged for further analysis and prediction tasks.

### 3.7. GraphML on the Enriched TF Cascades Knowledge Graph

Graph machine learning was used to score *candidate* TF→TF links; it did not alter the curated directed network used to build cascades. Predicted links and scores are provided separately (ROC AUC up to 0.97 with the Average operator).

The prediction of the next TF in a cascade was modeled as a supervised learning problem on top of TF node representations. We first generated a sub-graph where each node represents a transcription factor and the edges represent the relationship between the TFs. To perform similarity-based TF link prediction, we employed a network embedding technique called Node2Vec, which calculates node embeddings using random walk. The Node2Vec algorithm first runs random walks on the graph to obtain context pairs, which are then used to train a Word2Vec model. The resulting embeddings are learned in such a way to ensure that nodes that are close in the graph remain close in the embedding space. The parameters used for Node2Vec in our study were number of walks = 10, length of each random walk = 80, number of iterations = 10. We obtained 97,840 random walks.

Once the node embeddings are obtained, we can use them to perform link prediction using various binary operators such as Hadamard, L1, L2, and average. These operators are applied on the embeddings of the source and target nodes of each sampled edge to calculate link embeddings for the positive and negative edge samples. We used a random 75:25 split, with 75% of the data used for training the model and the remaining 25% used for testing. This ensures that the model is trained on a sufficiently large dataset while still having enough data left for testing. We then used a logistic regression classifier to train on the embeddings of the positive and negative examples to predict whether an edge between two nodes should exist or not. We evaluated the performance of the link classifier for each of the four operators on the training data with node embeddings calculated on the graph and selected the best classifier.

We considered four different operators, namely Hadamard, L1, L2, and Average operators, to generate link embeddings before classification (Table 11). Our experimental results showed that the best operator was the Average operator with an ROC AUC score of 0.971989. The choice of operator has implications for the quality of TF cascade link prediction and can be explored further to optimize the performance of the link prediction model.

## 4. Discussion

Limitations: Our analysis focuses on DNA-binding transcription factors and curated TF→TF regulatory actions. Co-factors, kinases, and chromatin remodelers that modulate TF activity were excluded by design to maintain a clear definition of cascade directionality. Extending the knowledge graph to include such regulators and non-cancer disease cohorts (beyond cBioPortal) is a planned extension.

Transcription factors are crucial regulators of gene expression and play a vital role in various cellular processes. In this study, we constructed a compendium of TF cascades using data extracted from the STRING database. Our analysis revealed 81,488 unique TF cascades, with the longest cascade consisting of 62 TFs, highlighting the intricate nature of TF interactions and the collaborative efforts of multiple TFs in gene regulation.

By employing centrality measurements, we identified the top 10 TFs with the highest regulatory influence. These TFs represent key players in the network and provide valuable insights for researchers interested in studying specific TFs. Understanding the regulatory roles and impact of these highly influential TFs is crucial for gaining deeper insights into their functional significance and their potential implications in disease processes.

One significant application of the PageRank and graphML results obtained from our analysis is the identification of potential drug targets among the highly ranked proteins. Discovering new drug targets is essential for developing effective treatments for cancer and other diseases. Leveraging the PageRank results, researchers can explore previously uninvestigated proteins as potential drug targets. This approach serves as a valuable checklist to guide the search for novel therapeutic interventions.

Furthermore, our pathway enrichment analysis uncovered significant enrichment of various pathways and functional categories, including those involved in cancer, development, differentiation, and cell signaling. These findings provide valuable insights into the dysregulation of TFs in disease states. The enriched pathways identified in this study may serve as potential targets for therapeutic intervention, offering new avenues for drug discovery and treatment strategies for diseases associated with TF dysregulation.

To ensure the accessibility and usability of our findings, we have made the TF cascades dataset, knowledge graph, and graphML methods publicly available. Additionally, we have developed a dedicated website for researchers to access and utilize this resource for their investigations. This resource serves as a valuable tool for researchers seeking to comprehend the complex network of TF interactions and their regulatory roles in cellular processes.

### 4.1. Clinical Perspective and Validation: How Identification of TF Cascades Can Identify Therapeutic Targets

Oncogenesis, post-operative wound healing, and hormonal aberrancies leading to metabolic derangements are often a result of improper TF cascade signaling [32,33,34]. Determination of TFs that bind to DNA to perform a particular function is based on the structure of motifs, of which 80% are helix turn helix (HTH), zinc finger (ZF), leucine zipper, forkhead, or helix loop helix (HLH) structures. Motif structures were earlier used to classify TFs, and each class proved a variable set of functions. Notably, HTH motifs were associated with hematopoietic precursor cells and leukemias [35], whereas Krüppel-like factors (KLFs) belong to multiple biological processes and are involved also in carcinogenesis [36]. On the other hand, FOXO genes that belong to the fork-head box family produce tumorsuppressor proteins that are often inactivated in human cancers.

Of the enriched pathways identified in our study, NANOG was seen to be of highest PageRank, followed by TP53. NANOG, a homeobox transcription factor essential for maintaining the pluripotency of Embryonic Stem Cells (ESCs), plays a critical role in tumorigenesis, progression, and metastasis in a variety of human cancers. The self-renewal and survival of cancer stem cells (CSCs), responsible for tumor initiation, are promoted by NANOG [37]. Creating targeted treatments for NANOG has drawn more attention in recent years. Understanding NANOG’s role may lead to it becoming a therapeutic target to halt cancer progression [38]. Recent studies have shown NANOG has demonstrated synergistic induction of naïve pluripotent cells through LIF signal transduction, causing increased STAT3, whereas NANOG limits the effect of KLF4 [39].

Previous studies have demonstrated that Krupple-like factor 5 (KLF5), a zinc-finger transcriptional factor, may be a potential therapeutic target for oxaliplatin-resistant CRC (colorectal carcinoma). KLF also activates gene promoters PDGF-A/-B, iNOS, PAI-1, and VEGF receptors, making it a target for cardiovascular remodeling [40]. In squamous cell carcinoma, KLF5 positively regulates Sox4 expression, and KLF5/Sox4 regulatory signaling facilitates tumorigenesis [41]. Furthermore, inhibition of KLF5 using siRNA or ML264 markedly decreased invasion and migration in EOC (epithelial ovarian cancer) cells [42]. However, genes regulated by KLF5 have not been well characterized with the advent of TF cascading; conventional pharmacotherapies may be combined with KLF5 inhibition in the future. Sex-determining region Y box 2 (SOX2) TF—significant in embryonic development—is a key regulator of CSCs in glioblastoma (GBM), a malignant brain tumor with a dismal prognosis. The current therapeutic target available for GBM is temozolomide (TMZ) [43]. Increased SOX2 expression increases the resilience to SOX2, whereas its inhibition improves drug action. SOX2 inhibition by miRNA145 decreases the chemoresistance and improves TMZ sensitivity [42].

MYC upregulates POU5F1(OCT4), attributed to the cell renewal process. MYC interacts with NANOG and SOX2 to maintain the balance between stemness and differentiation of cells. This is imperative, as previously the balance of transcription factor levels was not attributed to a combined regulatory mechanism; instead, it was considered to be controlled solely by POU5F1 [44]. There is existence of a stoichiometric effect for SOX2, POU5F1, NANOG, MYC and KLF4 in regulating POU5F1 transcription [45]. Our paper emphasizes the earlier known literature on TF functions and progressing the knowledge of TF interactions leads to deeper understanding. Our program would allow to uncover further TF cascades that could later reveal pertinent interactions.

### 4.2. Future Work

An intriguing finding in our dataset is the role of POU5F1, which is relatively understudied, despite being involved in 1,546 cascades. Further investigation can be conducted to assess the impact of specific TFs, such as POU5F1 and NANOG, in cancers occurring in the testis and lung. Analyzing their involvement and regulatory influence in these specific anatomical regions can provide insights into their potential contributions to cancer development and progression. In addition, the application of a smarter PageRank-like algorithm, incorporating disease-specific information and weighting incoming edges based on protein importance, can enhance the analysis and prioritize potential drug targets more accurately.

Furthermore, probabilistic models can be employed to approximate the likelihood of different outcomes following mutations or alterations in TF cascades. By incorporating specific data for each protein within the cascade, researchers can gain a deeper understanding of the potential consequences, such as cancer development, activation of other TFs, or gene dysregulation [46].

Going forward, TF research will enable precision medicine by obtaining a deeper understanding of transcription factor networks, creating personalized treatment approaches [47]. TF networks would also allow for drug repurposing [21], tissue engineering and expanding regenerative medicine by developing specific cell types from stem cells [48]. Transcription factors can be attractive drug targets [49]. With emerging CRISPR-Cas9 use for genome editing, the understanding of TF sites would enable cellular reprogramming [50]. Involving complex machine learning models on TFs would allow for predictions of its impact on health and disease. Moreover, due to the precise manipulations of gene expression through TFs, social, legal and ethical considerations must be taken into account in the future with careful oversight and guidelines [51].

## 5. Conclusions

This study contributes significantly to the understanding of the complex network of transcription factor (TF) interactions and their regulatory roles in cellular processes. By constructing a comprehensive compendium of 81,488 unique TF cascades, including the longest cascade comprising 62 TFs, and identifying highly influential TFs such as MYC, TP53, STAT3, and POU5F1, we provide a robust foundation for advancing research in transcriptional regulation. The integration of network analysis, PageRank, and graph machine learning has enabled the identification of potential drug targets, with POU5F1 emerging as a particularly promising candidate due to its prognostic relevance, as supported by Kaplan Meier survival analyses. These analyses revealed statistically significant survival disparities in patients with altered POU5F1 expression, highlighting its potential as a therapeutic target in cancers.

The pathway enrichment analysis further elucidated the biological mechanisms underlying TF cascades, identifying enriched pathways such as “Transcriptional misregulation in cancer” and “Th17 cell differentiation,” which are implicated in disease states. The publicly available TF cascades dataset, knowledge graph, and accompanying website serve as valuable resources for researchers, facilitating further exploration of TF interactions and their therapeutic implications. Future work should focus on validating these findings through experimental studies, refining predictive models with disease-specific data, and exploring the clinical translation of identified targets like POU5F1 to enhance precision medicine approaches.

## Figures and Tables

**Table 1:** Dictionary containing protein ID and gene name pairs.

Protein Stable ID	Gene Name
ENSP00000177694	*TBX21*
ENSP00000201031	*TFAP2C*
ENSP00000204517	*TFAP4*
ENSP00000216037	*XBP1*
ENSP00000217026	*MYBL2*
ENSP00000217086	*SALL4*
ENSP00000221452	*RELB*
ENSP00000222122	*DBP*
ENSP00000222598	*DLX5*
ENSP00000222726	*HOXA5*

**Table 2:** Result of PageRank.

TF	PageRank	Rank
NANOG	0.0169	1
TP53	0.0115	2
MYC	0.0113	3
AR	0.0100	4
SP7	0.0091	5
SNAI2	0.0085	6
SNAI1	0.0083	7
PPARG	0.0082	8
NEUROG3	0.0081	9
RUNX2	0.0079	10

**Table 3:** Betweenness centrality table.

ID	Degree	Betweenness Centrality
MYC	41	0.169411867
TP53	39	0.146407871
STAT3	40	0.137815540
SNAI1	18	0.069823653
NANOG	25	0.066742667
KLF4	15	0.055090028
AR	21	0.052755056
POU5F1	21	0.047753251
FOXM1	15	0.045555903
FOXO3	16	0.040374857

**Table 4:** Closeness centrality table.

ID	Degree	Closeness Centrality
MYC	41	0.353760708
STAT3	40	0.353404453
TP53	39	0.348145458
FOXO3	16	0.328586725
NANOG	25	0.328279347
SNAI1	18	0.326751045
POU5F1	21	0.315869147
FOXM1	15	0.315301547
FOXO1	15	0.312215856
AR	21	0.310833146

**Table 5:** Eigenvector centrality table.

ID	Degree	Eigenvector Centrality
STAT3	40	0.366485503
MYC	41	0.303674676
TP53	39	0.260577147
NANOG	25	0.240092313
POU5F1	21	0.218315088
FOXO3	16	0.173948202
SOX2	16	0.173898883
SNAI1	18	0.165160471
KLF5	12	0.154141463
FOXM1	15	0.153938826

**Table 6:** Survival type analysis.

Survival Type	Number of Patients	*p*-Value	q-Value
Disease Specific	6553	4.188 × 10^−3^	0.0168
Overall	6789	0.0318	0.0437
Progression Free	6787	0.0328	0.0437
Disease Free	3736	0.604	0.604

**Table 7:** Survival plot summary.

	Number of Cases, Total	Number of Events	Median Months Overall
Altered group	98	43	53.19
Unaltered group	6691	1946	91.99

**Table 8:** Result of pathway enrichment analysis.

Pathway	*p*-Value	Z-Score	Combined Score	Adjusted *p*-Value	Cascade_Value	Cascade
neuroactive ligand receptor interaction	0.4375	1.7699	1.4633	0.4375	6727	’STAT3’, ’JUNB’, ’JUN’, ’NANOG’, ’POU5F1’, ....
focal adhesion	0.371	2.2013	2.1829	0.3838	6928	’NCOA3’, ’MYC’, ’SNAI1’, ’NANOG’, ’POU5F1’, ’....’
mapk signaling pathway	0.37	2.2173	2.2043	0.37	7022	’EPAS1’, ’SOX2’, ’NANOG’, ’POU5F1’, ’SALL4’
purine metabolism	0.2848	3.0549	3.8371	0.3026	7284	’AR’, ’MYC’, ’SNAI1’, ’NANOG’, ’POU5F1’, ....
jak stat signaling pathway	0.2897	2.9943	3.7097	0.2982	4750	’LHX9’, ’NR5A1’, ’NR0B1’, ’POU5F1’, ’SALL4’, .....
axon guidance	0.2619	3.3748	4.5212	0.2916	7530	’STAT3’, ’SALL4’, ’NANOG’, ’POU5F1’, ’SNAI1’, ....
natural killer cell-mediated cytotoxicity	0.2673	3.2952	4.3481	0.2916	7530	’STAT3’, ’SALL4’, ’NANOG’, ’POU5F1’, ’SNAI1’, ....
tight junction	0.266	3.3149	4.39	0.2821	5310	’OTX2’, ’HMGA2’, ’TWIST1’, ’POU5F1’, ’SALL4’, ....
cell cycle	0.2216	4.1002	6.178	0.2574	7199	’STAT3’, ’NFATC2’, ’SOX2’, ’NANOG’, ’POU5F1’, ....
melanogenesis	0.2141	4.2667	6.577	0.2497	6966	’STAT6’, ’PPARG’, ’TP53’, ’NANOG’, ’POU5F1’, ....
gnrh signaling pathway	0.2064	4.4473	7.0166	0.2492	6779	’NCOA1’, ’MYC’, ’SNAI1’, ’NANOG’, ’POU5F1’, ....
pyrimidine metabolism	0.1831	5.0933	8.6465	0.2273	7199	’STAT3’, ’NFATC2’, ’SOX2’, ’NANOG’, ’POU5F1’, ....
apoptosis	0.1772	5.2851	9.1462	0.2215	7480	’EGR2’, ’CEBPB’, ’PPARG’, ’TP53’, ’NANOG’, ....
small cell lung cancer	0.1851	5.0324	8.4893	0.217	6528	’SNAI2’, ’RUNX2’, ’SP7’, ’SATB2’, ’NANOG’, ....
erbb signaling pathway	0.1851	5.0324	8.4893	0.217	6528	’SNAI2’, ’RUNX2’, ’SP7’, ’SATB2’, ’NANOG’, ....
chronic myeloid leukemia	0.1652	5.7154	10.2914	0.216	6956	’HOXA5’, ’SOX2’, ’NANOG’, ’POU5F1’, ’SALL4’, ....
glioma	0.1386	6.9379	13.71	0.2141	7314	’STAT3’, ’FOS’, ’SNAI1’, ’NANOG’, ’POU5F1’, ....
adherens junction	0.1652	5.7154	10.2914	0.2141	7314	’STAT3’, ’FOS’, ’SNAI1’, ’NANOG’, ’POU5F1’, ....
basal cell carcinoma	0.124	7.84	16.3687	0.2141	7314	’STAT3’, ’FOS’, ’SNAI1’, ’NANOG’, ’POU5F1’, ....
melanoma	0.1571	6.0432	11.1852	0.2141	7314	’STAT3’, ’FOS’, ’SNAI1’, ’NANOG’, ’POU5F1’, ....

**Table 9:** Graph summary (nodes).

Node Type	Description	Frequency
TFs	The final TF in a series of TF cascades is connected in a unidirectional manner to the preceding TFs within the cascade.	426
Pathways	Each terminal TF cascade will be linked to its corresponding pathway.	49
Total nodes		475

**Table 10:** Graph summary (edges).

Edges	Description	Frequency
All TFs	Weights for connections between transcriptions in each cascade are assigned as 1, and for the entire graph, it would be the sum of all connections in the dataset.	5209
All pairs of TFs and pathways	Weight information on *p*-value, Z-score, and combined score for all available pathways. The pairs without connection are marked with 0	33,418
Total edges		38,627

**Table 11:** TF graph ML ROC-AUC scores.

Operator	ROC-AUC Score
Hadamard	0.800292
L1	0.798369
L2	0.803425
Average	0.971989

## Data Availability

The data presented in this study are available in the article and its supplementary materials. The TF cascades dataset and knowledge graph are publicly accessible at https://sonishsivarajkumar.github.io/TFCascades/ (accessed on 1 January 2020).

## Supplementary Materials

The following supporting information can be downloaded at: https://www.mdpi.com/article/10.3390/genes16121430/s1. Figure S1. TP53 alteration frequency across cancer types: Bar plot showing the percentage of cases with TP53 gene alterations in multiple cancer cohorts, sourced from cBioPortal. Table S1. Directed TF→TF edge retention parameters: Key settings and thresholds used for filtering and constructing the directed transcription factor-to-transcription factor edge network, including source databases, score cut-offs, and cascade enumeration criteria. Supplementary Note 1: Web app usage guide.

## References

[1] Lambert S.A., Jolma A., Campitelli L.F., Das P.K., Yin Y., Albu M., Chen X., Taipale J., Hughes T.R., Weirauch M.T. (2018). The Human Transcription Factors. Cell.

[2] Jamal M., Bangash H.I., Habiba M., Lei Y., Xie T., Sun J., Wei Z., Hong Z., Shao L., Zhang Q. (2021). Immune dysregulation and system pathology in COVID-19. Virulence.

[3] Lee T.I., Young R.A. (2013). Transcriptional Regulation and Its Misregulation in Disease. Cell.

[4] Fong H., Hohenstein K.A., Donovan P.J. (2013). Regulation of Self-Renewal and Pluripotency by Sox2 in Human Embryonic Stem Cells. Stem Cells.

[5] Takahashi K., Yamanaka S. (2016). A Decade of Transcription Factor-Mediated Reprogramming to Pluripotency. Nat. Rev. Mol. Cell Biol..

[6] Naika M., Shameer K., Mathew O.K., Gowda R., Sowdhamini R. (2013). STIFDB2: An updated version of plant stress-responsive transcription factor database with additional stress signals, stress-responsive transcription factor binding sites and stress-responsive genes in Arabidopsis and rice. Plant Cell Physiol..

[7] https://www.nature.com/scitable/topicpage/transcription-factors-and-transcriptional-control-in-eukaryotic-1046/.

[8] Alvarez M.J., Shen Y., Giorgi F.M., Lachmann A., Ding B.B., Ye B.H., Califano A. (2016). Functional characterization of somatic mutations in cancer using network-based inference of protein activity. Nat. Genet..

[9] Surget S., Khoury M.P., Bourdon J.C. (2013). Uncovering the role of p53 splice variants in human malignancy: A clinical perspective. Onco Targets Ther..

[10] Cerami E., Gao J., Dogrusoz U., Gross B.E., Sumer S.O., Aksoy B.A., Jacobsen A., Byrne C.J., Heuer M.L., Larsson E. (2012). The cBio Cancer Genomics Portal: An Open Platform for Exploring Multidimensional Cancer Genomics Data. Cancer Discov..

[11] Karamouzis M.V., Papavassiliou K.A., Adamopoulos C., Papavassiliou A.G. (2018). Targeting Androgen/Estrogen Receptors Crosstalk in Cancer. Trends Cancer.

[12] Wu Z., Li W., Liu G., Tang Y. (2018). Network-Based Methods for Prediction of Drug-Target Interactions. Front. Pharmacol..

[13] Roy R., Marakkar S., Vayalil M.P., Shahanaz A., Anil A.P., Kunnathpeedikayil S., Rawal I., Shetty K., Shameer Z., Sathees S. (2022). Drug-Food Interactions in the Era of Molecular Big Data, Machine Intelligence, and Personalized Health. Recent Adv. Food Nutr. Agric..

[14] Olayan R.S., Ashoor H., Bajic V.B. (2018). DDR: Efficient Computational Method to Predict Drug-Target Interactions Using Graph Mining and Machine Learning Approaches. Bioinformatics.

[15] Thafar M.A., Olayan R.S., Albaradei S., Bajic V.B., Gojobori T., Essack M. (2022). Affinity2Vec: Drug-Target Binding Affinity Prediction Through Representation Learning, Graph Mining, and Machine Learning. Sci. Rep..

[16] Szklarczyk D., Morris J.H., Cook H., Kuhn M., Wyder S., Simonovic M., Santos A., Doncheva N.T., Roth A., Bork P. (2016). The STRING Database in 2017: Quality-Controlled Protein-Protein Association Networks, Made Broadly Accessible. Nucleic Acids Res..

[17] Palasca O., Santos A., Stolte C., Gorodkin J., Jensen L.J. (2018). TISSUES 2.0: An Integrative Web Resource on Mammalian Tissue Expression. Database.

[18] UniProt Consortium (2019). UniProt: A Worldwide Hub of Protein Knowledge. Nucleic Acids Res..

[19] Jacobsen A., Dogrusoz U., Aksoy B.A., Cerami E., Sander C., Gross B. (2015). cgdsr: R-Based API for Accessing the MSKCC Cancer Genomics Data Server (CGDS). R Package Version. https://github.com/cBioPortal/cgdsr.

[20] Masterton G., Olsson E.J. (2018). PageRank’s Ability to Track Webpage Quality: Reconciling Google’s Wisdom-of-Crowds Justification with the Scale-Free Structure of the Web. Heliyon.

[21] Shameer K., Readhead B., Dudley J.T. (2018). Computational and Experimental Advances in Drug Repositioning for Accelerated Therapeutic Stratification. Curr. Top. Med. Chem..

[22] Glicksberg B.S., Li L., Badgeley M.A., Shameer K., Kosoy R., Beckmann N.D., Pho N., Hakenberg J., Ma M., Ayers K.L. (2016). Comparative Analyses of Population-Scale Phenomic Data in Electronic Medical Records Reveal Race-Specific Disease Networks. Bioinformatics.

[23] Kuleshov M.V., Jones M.R., Rouillard A.D., Fernandez N.F., Duan Q., Wang Z., Koplev S., Jenkins S.L., Jagodnik K.M., Lachmann A. (2016). Enrichr: A Comprehensive Gene Set Enrichment Analysis Web Server 2016 Update. Nucleic Acids Res..

[24] Thissen D., Steinberg L., Kuang D. (2002). Quick and Easy Implementation of the Benjamini-Hochberg Procedure for Controlling the False Discovery Rate in Multiple Comparisons. J. Educ. Behav. Stat..

[25] Hu W., Yang Y., Li X., Zheng S. (2018). Pan-organ transcriptome variation across 21 cancer types. Front. Pharmacol..

[26] Yacoumatos C., Bragaglia S., Kanakia A., Svangård N., Mangion J., Donoghue C., Weatherall J., Khan F.M., Shameer K. (2021). TrialGraph: Machine Intelligence Enabled Insight from Graph Modelling of Clinical Trials. arXiv.

[27] Hagberg A., Schult D., Swart P. Exploring Network Structure, Dynamics, and Function Using NetworkX. Proceedings of the 7th Python in Science Conference.

[28] Gaudelet T., Day B., Jamasb A.R., Soman J., Regep C., Liu G., Hayter J.B.R., Vickers R., Roberts C., Tang J. (2021). Utilizing graph machine learning within drug discovery and development. Brief Bioinform..

[29] Grover A., Leskovec J. node2vec: Scalable Feature Learning for Networks. Proceedings of the KDD ’16: 22nd ACM SIGKDD International Conference on Knowledge Discovery and Data Mining.

[30] Stower H. (2013). Gene expression: Super enhancers. Nat. Rev. Genet..

[31] Chew J.L., Loh Y.H., Zhang W., Chen X., Tam W.L., Yeap L.S., Li P., Ang Y.S., Lim B., Robson P. (2005). Reciprocal transcriptional regulation of Pou5f1 and Sox2 via the Oct4/Sox2 complex in embryonic stem cells. Mol. Cell Biol..

[32] Vishnoi K., Viswakarma N., Rana A., Rana B. (2020). Transcription Factors in Cancer Development and Therapy. Cancers.

[33] Imodoye S.O., Adedokun K.A., Muhammed A.O., Bello I.O., Muhibi M.A., Oduola T., Oyenike M.A. (2021). Understanding the Complex Milieu of Epithelial-Mesenchymal Transition in Cancer Metastasis: New Insight Into the Roles of Transcription Factors. Front Oncol..

[34] Shiah J.V., Johnson D.E., Grandis J.R. (2023). Transcription Factors and Cancer: Approaches to Targeting. Cancer J..

[35] Shima Y., Kitabayashi I. (2011). Deregulated transcription factors in leukemia. Int. J. Hematol..

[36] McConnell B.B., Yang V.W. (2010). Mammalian Krüppel-Like Factors in Health and Diseases. Physiol. Rev..

[37] Gong S., Li Q., Jeter C.R., Fan Q., Tang D.G., Liu B. (2015). Regulation of NANOG in cancer cells. Mol. Carcinog..

[38] Vasefifar P., Motafakkerazad R., Maleki L.A., Najafi S., Ghrobaninezhad F., Najafzadeh B., Alemohammad H., Amini M., Baghbanzadeh A., Baradaran B. (2022). Nanog, as a key cancer stem cell marker in tumor progression. Gene.

[39] Stuart H.T., van Oosten A.L., Radzisheuskaya A., Martello G., Miller A., Dietmann S., Nichols J., Silva J.C. (2014). NANOG amplifies STAT3 activation and they synergistically induce the naive pluripotent program. Curr. Biol..

[40] Nagai R., Suzuki T., Aizawa K., Shindo T., Manabe I. (2005). Significance of the Transcription Factor KLF5 in Cardiovascular Remodeling. J. Thromb. Haemost..

[41] Li Q., Dong Z., Zhou F., Cai X., Gao Y., Wang L.W. (2014). Krüppel-like factor 5 promotes lung tumorigenesis through upregulation of Sox4. Cell Physiol. Biochem..

[42] Siraj A.K., Pratheeshkumar P., Divya S.P., Parvathareddy S.K., Alobaisi K.A., Thangavel S., Siraj S., Al-Badawi I.A., Al-Dayel F., Al-Kuraya K.S. (2020). Krupple-Like Factor 5 is a Potential Therapeutic Target and Prognostic Marker in Epithelial Ovarian Cancer. Front. Pharmacol..

[43] Garros-Regulez L., Garcia I., Carrasco-Garcia E., Lantero A., Aldaz P., Moreno-Cugnon L., Arrizabalaga O., Undabeitia J., Torres-Bayona S., Villanua J. (2016). Targeting SOX2 as a Therapeutic Strategy in Glioblastoma. Front. Oncol..

[44] Das B., Pal B., Bhuyan R., Li H., Sarma A., Gayan S., Talukdar J., Sandhya S., Bhuyan S., Gogoi G. (2019). MYC Regulates the HIF2*α* Stemness Pathway via Nanog and Sox2 to Maintain Self-Renewal in Cancer Stem Cells versus Non-Stem Cancer Cells. Cancer Res..

[45] Wang Z., Oron E., Nelson B., Razis S., Ivanova N. (2012). Distinct Lineage Specification Roles for NANOG, OCT4, and SOX2 in Human Embryonic Stem Cells. Cell Stem Cell.

[46] Shameer K., Johnson K.W., Glicksberg B.S., Dudley J.T. (2017). Translational Bioinformatics in the Era of Real-Time Biomedical, Health Care and Wellness Data Streams. Brief. Bioinform..

[47] Tan A., Huang H., Zhang P., Li S. (2019). Network-based cancer precision medicine: A new emerging paradigm. Cancer Lett..

[48] Wang H., Yang Y., Liu J., Qian L. (2021). Direct cell reprogramming: Approaches, mechanisms and progress. Nat. Rev. Mol. Cell. Biol..

[49] Hazelett D.J., Lakeland D.L., Weiss J.B. (2009). Affinity Density: A novel genomic approach to the identification of transcription factor regulatory targets. Bioinformatics.

[50] Pandelakis M., Delgado E., Ebrahimkhani M.R. (2020). CRISPR-Based Synthetic Transcription Factors In Vivo: The Future of Therapeutic Cellular Programming. Cell Syst..

[51] Moradi S., Mahdizadeh H., Šarić T., Kim J., Harati J., Shahsavarani H., Greber B., Moore J.B. (2019). Research and therapy with induced pluripotent stem cells (iPSCs): Social, legal, and ethical considerations. Stem Cell Res. Ther..

