# Supplementary material for: Generation of a Compendium of Transcription Factor Cascades and Identification of Potential Therapeutic Targets Using Graph Machine Learning
Source: Genes (Basel). 2025 Nov 30;16(12):1430. doi: 10.3390/genes16121430 (PMC12733007; doi:10.3390/genes16121430)
Supplement: Supplementary file 1 [file genes-16-01430-s001.zip › genes-3765696-supplementary.pdf]

Supplementary Materials for

Generation of a Compendium of Transcription Factor Cascades and Identification of Potential Therapeutic Targets using Graph Machine Learning

Supplementary Figure S1. TP53 alteration frequency across cancer types

Bar plot showing alteration frequency (%) of *TP53* across multiple cancer cohorts from cBioPortal.

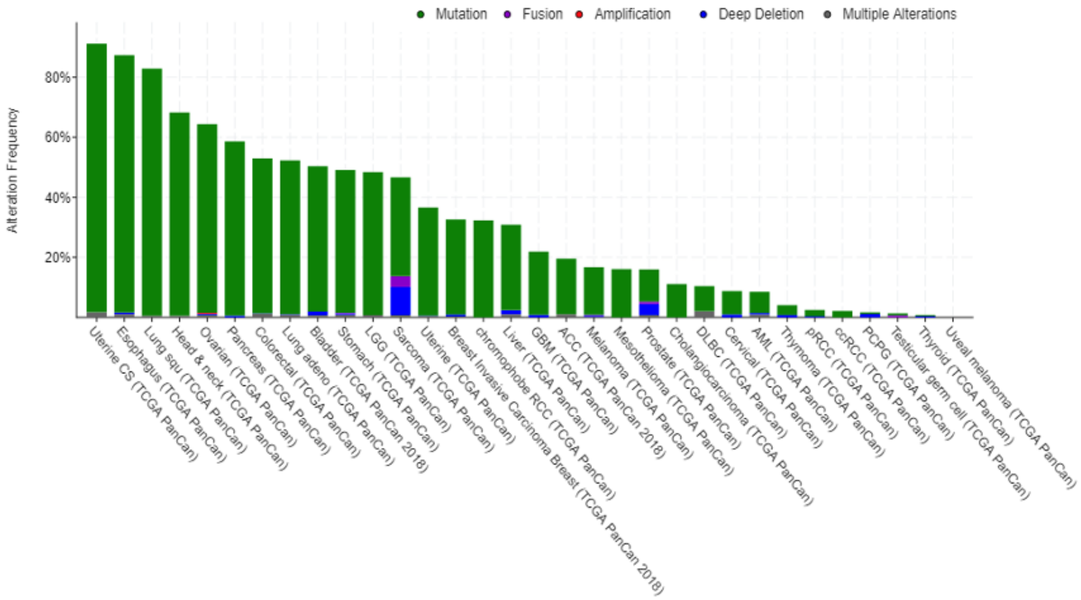

Supplementary Note 1. Web app usage guide

Access

The resource is available via the Streamlit app (https://tfcascades.streamlit.app/) and a static GitHub Pages mirror (https://sonishsivarajkumar.github.io/TFCascades/). If the Streamlit app is idle, the static mirror provides immediate browsing of EDA summaries.

Browse cascades

Use the *Cascades* tab to filter by cascade length (level) and to download selected cascades as CSV.

Centrality and rankings

The *Centrality* tab lists top TFs by PageRank/eigenvector/closeness/betweenness computed on the directed TF network. Click any TF to view the cascades it participates in.

Predicted links

The *Predictions* tab exposes GraphML link scores (Average operator). These predictions are provided for hypothesis generation and are not merged into the curated network.

Supplementary Table S1. Directed TF→TF edge retention parameters

Parameter	Value
STRING dataset	v11 “actions” (Human)
Edge orientation	Regulator → target (activation/inhibition)
Score threshold	Combined score $\geq$ 700
Cascade enumeration	Directed simple paths, DFS; cycles pruned; dedup node sequences
Outputs	Edge list with scores; path parameters
